# On the Influence of Syphilis in the Production of Tubercle

**Published:** 1870-06

**Authors:** 


### On the Influence of Syphilis in the Production of Tubercle.

The fact of the awakening of a dormant predisposition to tuberculosis by syphilis Dr. Lebert has frequently observed, as well as the power of syphilis to produce tuberculosis where no predisposition had existed. The microscopical appearance of the neoplasm, produced by syphilis in the lung, are not at all distinctive; they do not resemble gummy tumors, and in fact cannot be differentiated from the same lieson produced by the cachexia of tuberculosis; they may be regarded as a perversion of nutrition by profound infection of the organism, the morbid processes in either instance producing very similar results. (Well-defined gummy tumor, however, is sometimes found in the lungs.) Having established the fact of the development of tuberculosis of the lungs and and glands by syphilis, attention is called to the necessity, in cases of phthisis, of the recognition of the syphilitic cause, if it exist, and the institution of the treatment indicated. Dr. Lebert has himself seen marked amelioration and even permanent recovery in such cases under the influence of proper medication.

The treatment of one particular case was by the inunction of 16 grains of mercurial ointment; at first once dayly, subsequently twice each day. The mode of inunction directed is somewhat peculiar: "A piece of bladder is to be moistened and then allowed to dry only so far that it may still remain soft; this bladder, when filled with cotton wool, constitutes a tampon on which the ointment is to be placed and by means of which it is to be rubbed in during about fifteen or twenty minutes at a time. The inunctions must be made at different places alternately, so as not cause an eruption at any of the places." In the use of mercury for constitutional syphilis, Lebert very much prefers the inunction to other modes of administration, as it acts more efficiently, is less liable to disturb digestion, or to produce those unpleasant symptoms of ptyalism which though not syphilitic very much annoy the patient. He adverts to the fact that inunction at one period fell into disrepute, by reason of the fact that physicians when using the treatment confined the patients to very warm atmospheres and low diet. He regards salivation as injurious, and thinks that as anæmia and impaired nutrition are unfailing concomitants of syphilis, low diet and the debilitating influences of high temperatures are very prejudicial and to be avoided. He summarizes his views as to admintration of mercury and the treatment of constitutional syphilis as follows: 1. Mercury is preferable administered in the form of inunction to that of hypodermic injection or by the mouth. The quantity daily required by the method is thirty-two grains. 2. Combined with mercurial unction, an invigorating and nourishing diet is necessary, avoiding any digestive disturbance. The patient should not be confined to his room, but may be active in out-door pursuits, if he avoids cold and wet. 3. Ptyalism is always to be avoided; this is accomplished by washing the mouth with common water, by frequent bathing and by the use of chlorate of potash both internally and as a gargle when the first signs are noticeable. 4. Avoiding unnecessary fasting and perspiration, the course can be pursued without attracting notice, can be continued indefinitely, and repeated in case of relapse. 6. In inveterate cases of constitutional syphilis, good effects follow the combined internal administration of iodide of potassium with the inunction.—*Med. Times and Gazette,—American Jour. Syphilography and Derm.*

